# NURSE WAGES AND NURSING HOME FINANCIAL PERFORMANCE

**DOI:** 10.1093/geroni/igae098.0535

**Published:** 2024-12-31

**Authors:** Justin Lord, Akbar Ghiasi, Shivani Gupta, Rohit Pradhan

**Affiliations:** Louisiana State University Shreveport, Shreveport, Louisiana, United States; University of the Incarnate Word, San Antonio, Texas, United States; University of Houston - Clear Lake, Houston, Texas, United States; Texas State University, San Marcos, Texas, United States

## Abstract

Nursing home (NH) care is labor intensive, with staff compensation being the most significant expense. Balancing higher wages to attract qualified nurses against the allocation of resources to other facility areas is crucial. This study examined the impact of nurse wages on NH financial performance. This study used secondary datasets such as the Payroll Based Journals, Medicare cost reports. and Care Compare (2017-2022). It included all U.S. NHs certified by the Centers for Medicare and Medicaid Services (CMS). Analytic data comprised 80,244 facilities, averaging 13,374 unique NHs per year. We modeled the data using multivariate linear regression with two-way fixed effects (year and state). The operating margin at the facility level served as the dependent variable, with inflation-adjusted nurse wages as the main variable of interest. Separate models for registered nurses (RNs), licensed practical nurses (LPNs), and certified nursing assistants (CNAs) showed that a 10% wage increase correlated with operating margin decreases of 7.3%, 9.6%, and 11.5%, respectively (p<.01). Positive operating margins were associated with for-profit status, chain affiliation, larger facilities, higher occupancy, and a greater proportion of Medicare residents, while higher uninsured rates and Medicare Advantage penetration negatively impacted margins. These findings underscore the necessity for striking a strategic balance between nurse compensation and NH financial health. Given the important role nurses play in NHs, and amidst an acute staffing shortage, our study emphasizes the need for policy measures that support both competitive nurse wages and the sustainability of NH operations, ensuring high-quality care and long-term viability.

